# Radiofrequency sacroplasty as a pain treatment for symptomatic sacral destruction due to prostate metastasis: a case report and a review of the literature

**DOI:** 10.1093/jscr/rjae733

**Published:** 2025-03-13

**Authors:** Julian Ramin Andresen, Harald K Widhalm, Reimer Andresen

**Affiliations:** Department of Orthopedics and Trauma Surgery, Medical University of Vienna, Währinger Gürtel 18-20, 1090 Vienna, Austria; Department of Orthopedics and Trauma Surgery, Medical University of Vienna, Währinger Gürtel 18-20, 1090 Vienna, Austria; Institute of Diagnostic and Interventional Radiology/Neuroradiology, Westkuestenklinikum Heide, Academic Teaching Hospital of the Universities of Kiel, Luebeck and Hamburg, Esmarchstraße 50, 25746 Heide, Germany

**Keywords:** bone metastases, interventional pain therapy, pathological fracture, radiofrequency sacroplasty, sacrum

## Abstract

In progressive malignant diseases such as prostate cancer, metastatic bone destruction is a frequent cause of chronic, debilitating pain. Pathological fractures of the sacrum can lead to complete immobility in patients. Cement augmentation of the sacrum has proven effective in treating sacral insufficiency fractures, resulting in significant pain reduction and improved mobility. In recent years, this technique has also been applied to pathological fractures of the sacrum. We present a case of successful treatment of prostate cancer-induced sacral bone infiltration and destruction using advanced radiofrequency sacroplasty. Literature reports describe individual cases of sacral bone destruction, where cement augmentation using various methods has led to substantial pain relief and improved overall outcomes.

## Introduction

In malignant diseases such as breast cancer, prostate cancer, bronchial carcinoma, renal cell carcinoma, thyroid cancer, multiple myeloma, and lymphoma, metastases in the axial skeleton are common in advanced stages of the disease. Osseous metastases are the most common malignant bone disease in adults. They have a negative impact on the quality of life and worsen the patient’s prognosis [1]. Even taking all treatment options into account, the 5-year survival rate for patients with metastatic prostate cancer is about 30% [2]. If the sacrum is affected with destruction and subsequent pathological fracture, disabling pain as a cardinal syndrome in the lower lumbar spine and pelvic region is the main symptom. Conservative treatment with confinement to bed and analgesic therapy frequently does not achieve a satisfactory reduction in pain and can also lead to the development of decubitus ulcers, venous thrombosis and pulmonary artery embolism as well as pneumonia [3]. Percutaneous cement augmentation of the sacrum is increasingly being used successfully for pain therapy in osteoporosis-related insufficiency fractures [3–5] and metastasis-related destructions [6–9]. The aim of the interventional therapy in this palliative context is the fast reduction of pain increasing the mobility and quality of life of the patients and preventing progressive destruction of the affected bone. Thus, cement augmentation of the sacrum can facilitate the further necessary treatment such as chemo-, hormone-, and radiotherapy. Radiofrequency sacroplasty (RFS) is considered a further improvement of percutaneous cement augmentation in osteoporotic fractures allowing reliable and rapid pain reduction [10].

For the first time, we report on the successful, complication-free treatment of a metastasis-induced destruction of the sacrum with RFS due to prostate cancer.

## Case report

**Figure 1:**
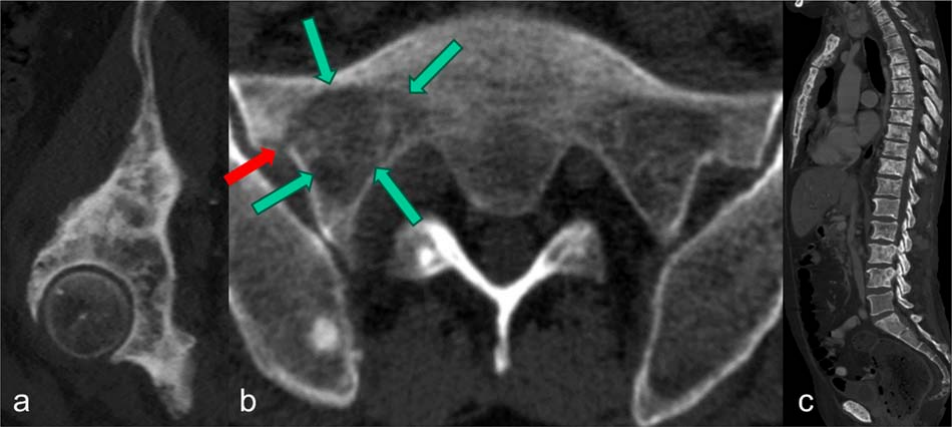
In the sagittal reformation of the right hip (a), in the axial CT section of the right ilium (b) and in the sagittal reformation of the spine (c) osteoblastic metastases of the known prostate carcinoma are visible. In (b), there is also an osseous defect zone (marked with green arrows) in the right lateral mass of the sacrum with a pathological fracture (marked with red arrow) in the area of the lateral cortical border.

We present the case of a 60-year-old man diagnosed with osseous metastases and extraskeletal involvement from prostate cancer. Despite undergoing hormone therapy, progression of the bone metastases was observed. The patient also experienced worsening low back pain, which severely limited his daily activities and caused partial immobility. Despite receiving analgesic treatment in accordance with WHO guidelines, he rated his pain at 9 on a visual analog scale (VAS), with 10 being the maximum. At this stage, he required the use of a walking aid. In addition to other findings in the pelvic bones and spine (Fig. 1a and c), CT images revealed a metastasis with a subsequent pathological fracture in the lateral portion of the sacrum on the right side (Fig. 1b). Following a discussion at our regular interdisciplinary oncological conference, radiation therapy was recommended. To alleviate the patient’s persistent and disabling pain and prevent further bone destruction, it was also decided to treat the sacral fracture with cement augmentation.

**Figure 2:**
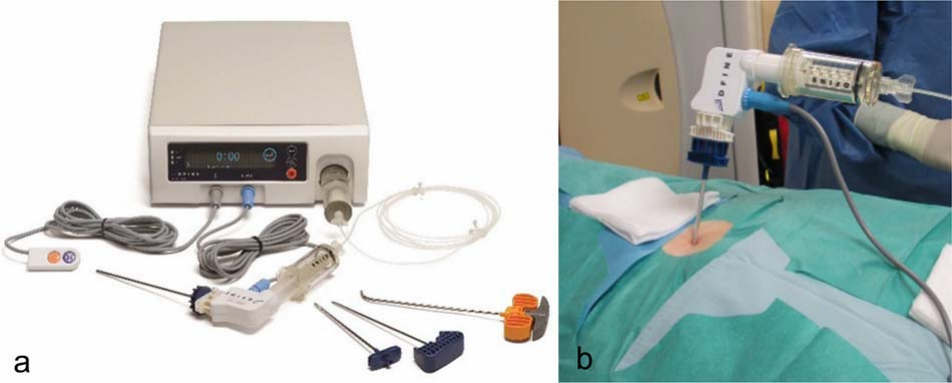
In a, image of the StabiliT® Vertebral Augmentation System (DFine Europe) with remote control, the cement applicator and the bone needles or osteotome (from left to right) as well as the control unit in the background. In (b), image of the connected application system.

**Figure 3:**
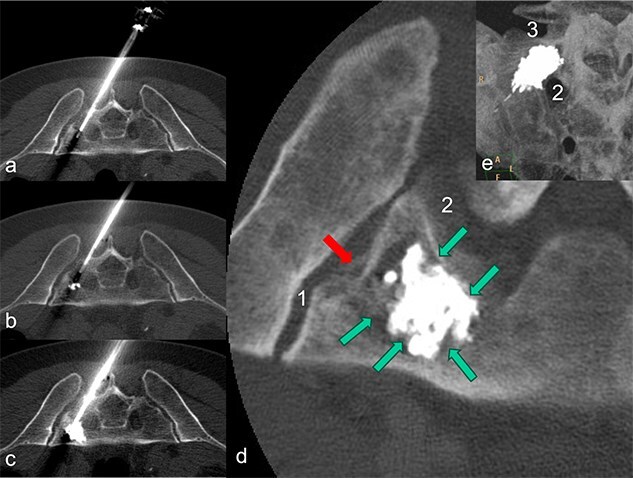
The patient is lying prone in the CT. The needle of the application system (a) was inserted from the dorsal side via the short axis. During the cement insertion, the successive, discontinuous filling of the metastasis-related defect zone in the right lateral mass of the sacrum is shown in (a) to (b). The highly viscous cement activated by radio frequency enables precise augmentation while simultaneously blocking a leak. In (d), the inserted PMMA cement is placed centrally in the defect zone as a cement seal (marked by green arrows); there is no cement leakage through the pathological fracture (marked by a red arrow) in the direction of the sacroiliac artery (1) and the neuroforamen (2). A MIP-reformation (d) shows the cement filling, whereby here too there is no leakage in the direction of the neuroforamen (2) and the adjacent disc space (3).

After discussing the treatment options and outcomes with the patient, we opted for the latest technique involving radiofrequency-activated ultrahigh-viscosity cement. The procedure was performed under intubation anesthesia with continuous anesthesiological monitoring. The patient was positioned prone in the CT scanner, and as part of routine practice, a single-shot antibiotic (cefazoline 2 g i.v.) was administered during the procedure. Based on the available access routes [11], the short axis of the sacrum was selected as the optimal entry point. Using a Jamshidi needle, access to the fracture zones was achieved dorsally. The cavity in the sacrum was filled with radiofrequency-activated bone cement (polymethylmethacrylate, PMMA; ER2 Bone Cement, DFine Europe) through the StabiliT Vertebral Augmentation System (DFine Europe) (Fig. 2a and b). The defect area was filled in a controlled, stepwise manner using single-slice CT guidance (Fig. 3a–c), with a total of 7 ml of PMMA cement.

Final imaging with low-dose, thin-slice spiral CT (Philips Brilliance TM CT 16-slice Big Bore, Philips GmbH DACH Healthcare, Hamburg, Germany) showed a well-centered cement distribution without leakage into the neural foramina, sacroiliac joint, adjacent disc space, or visceral surface of the sacrum (Fig. 3d and e). Post-procedurally, the patient experienced a reduction in pain to a VAS score of 4. His mobility, previously limited by pain, improved significantly, allowing for discharge 2 days after the procedure. By the time radiation therapy commenced, ~2 weeks after the RFS, the patient’s back pain had decreased to a VAS score of 2, and he was able to walk unaided. Any intermittent pain in the pelvic bones and spine was managed with NSAIDs at that time.

## Literature research

In PubMed, EMBASE, Google Scholar, and WorldCat, the following keywords were searched: interventional pain therapy, painful osseous sacral metastases, percutaneous sacroplasty, and sacral cement augmentation.

Table 1 summarizes the cases found after treatment of metastasis-related bony sacral destruction using different methods of cement augmentation.

**Table 1:** Literature review of sacroplasties performed in patients with metastatic or tumor-related osseous infiltrations

Author	Number of patients	Primary tumor	Method	Leakage	Follow-up, Pain reduction
Yoong *et al*. [6]	1	1× prostate carcinoma	VSP	None	2 months, yes
Tian *et al.* [7]	10	4× bronchial carcinoma 3× hepatocellular carcinoma 3× unknown cases	VSP	Yes, in three cases without clinical relevance	Until shortly after the intervention, yes (90% of cases)
Tian *et al.* [8]	35	14× bronchial carcinoma 8× thyroid carcinoma 6× hepatocellular carcinoma 7× unknown cases	VSP	Yes, Extraosseous leakage in 12 out of 35 cases, but without a relevant clinic	12 months, yes
Tian *et al.* [9]	126	44× bronchial carcinoma 22× liver carcinoma 21× thyroid carcinoma 16× breast cancer 10× prostate carcinoma 8× renal cell carcinoma 2× hemangiosarcoma 1× gallbladder carcinoma 1× colon carcinoma 1× parotid carcinoma	75 VSP and 51 VSP plus RFA	Yes, a leakage was found in 30 cases, Of these, 3 were symptomatic	12 months, yes in 76% of the VSP group and 90.2% in the VSP plus RFA group
Dehdashti *et al*. [12]	3	unknown	VSP	Yes, one leakage, asymptomatic	2 days, yes
Hierholzer *et al*. [13]	1	1× bronchial carcinoma	VSP	Yes, asymptomatic	2 months, yes
Butler *et al.* [14]	1	1× multiple myeloma	VSP	Yes, asymptomatic	2 weeks, yes
Uemura *et al.* [15]	1	1× hepatocellular carcinoma	VSP	None	10 days, yes
Masala *et al.* [16]	1	1× bronchial carcinoma	VSP	None	4 months, yes
Atalay *et al.* [17]	1	1× sacral hemangioma	BSP	None	1 month
Valencia-Anguita *et al*. [18]	1	1× breast cancer	VSP	None	6 months, yes
Wee *et al*. [19]	2	1× multiple myeloma 1× renal cell carcinoma	VSP	Yes, in one case, asymptomatic	3–6 months, yes
Zhang *et al*. [20]	2	1× bronchial carcinoma 1× lymphoma	VSP	Yes, in one case, asymptomatic	12–20 weeks, yes
Georgy [21]	11	11× metastases of different primary tumors	VSP	Yes, three cases with mild symptoms	4 weeks, yes
Basile *et al*. [22]	8	8× multiple myeloma	VSP	Yes, in one case, asymptomatic	3–27 months, yes
Toro *et al.* [23]	1	1× hepatocellular carcinoma	VSP	None	2 months, yes
Lüdtke *et al*. [24]	1	1× rectal carcinoma	BSP	None	2 days, yes
Shah *et al.* [25]	5	5× metastases of different primary tumors	BSP	Yes, in one case, slightly symptomatic	2 days, yes
Sun *et al.* [26]	7	2× breast cancer 3× lung carcinoma 1× renal carcinoma 1× hepatocellular carcinoma	VSP	A leakage without significant clinical	3–6 months, yes
Kortman *et al*. [27]	39	11× breast cancer 11× multiple myeloma 2× lymphoma 1× hemangioma 1× cystic lesion 13× metastases of unproven primary tumors	VSP	None	30.5 months on average, yes, except for 4 cases
Pereira *et al.* [28]	49	19× breast cancer 5× lung carcinoma 4× thyroid carcinoma 2× endometrial carcinoma 1× esophageal carcinoma 1× rectal carcinoma 2× urinary bladder carcinoma 8× unknown primary tumor 4× multiple myeloma 1× hemangioma 1× chondrosarcoma 1× chordoma	VSP	Yes, symptomatic in four cases	30 days, yes in 85% of cases
Andresen *et al*. [29]	10	4× multiple myeloma 2× bronchial carcinoma 1× breast carcinoma 1× hepatocellular carcinoma 1× renal cell carcinoma 1× urothelial carcinoma	BSP	None	6 months, yes
Dmytriw *et al.* [30]	1	1× multiple myeloma	VSP	None	24 months, yes
Own case	1	1× prostate carcinoma	RFS	None	2 weeks, yes

The following abbreviations are used: VSP = vertebro sacroplasty, BSP = balloon sacroplasty, RFA = radiofrequency ablation, and RFS = radiofrequency sacroplasty.

In total, Table 1 contains 318 treated patients from 23 publications plus our own case.

Of these, metastasis-related or primary bone destruction was found: 75× bronchial carcinoma, 50× breast carcinoma, 50× unknown cases, 35× hepatocellular carcinoma, 33× thyroid carcinoma, 30× multiple myeloma, 12× prostate carcinoma, 11× renal cell carcinoma, 6× hemangiocarcinoma/hemangioma/cystic lesion, 3× colon carcinoma, 3× lymphoma, 2× endometrial carcinoma, 2× urinary bladder carcinoma, 1×urothelial carcinoma, 1× gallbladder carcinoma, 1× parotid carcinoma, 1× esophageal carcinoma, 1× chondrosarcoma, and 1× chordoma.

In the case of clinically irrelevant complications, there is generally a clear improvement in terms of pain reduction.

## Discussion

In recent years, there has been a growing number of literature on the treatment of painful insufficiency fractures and metastasis-related osseous destruction of the sacrum using various cement augmentation techniques [3–33]. These techniques share a common mechanism of action, where the injected PMMA cement stabilizes micromovements, typically resulting in rapid, significant, and sustained pain relief [29, 33]. The selection of the surgical approach depends on the fracture location, as well as the position and size of the osseous destruction, with specific attention to the axial plane, where approaches via the short or transiliac axis can be performed with minimal complications [6, 9, 11, 13, 19, 20, 27]. Adequate visualization of the bone structures is crucial for the safe insertion of the needle system, especially in cases with osteopenic bone texture or extensive osseous destruction. CT-guided intervention [5, 6, 10, 11, 13–15, 19, 24, 29] is superior to fluoroscopy-guided procedures [7–9, 12, 17, 18, 20, 22, 25, 28, 30] in minimizing complications related to needle misplacement and cement leakage [32]. In certain cases, a combination of both techniques may be beneficial [16, 21, 23, 26, 27].

The risk of cement leakage, which is not always asymptomatic, is higher with VSP than with BSP or RFS [33]. Compared to conventional cement augmentation (VSP), the PMMA cement activated by radiofrequency (RFS) enables a longer working time of up to 30 minutes with an unchanging high viscosity. Another advantage is the remote-controlled hydraulic application system (StabiliT® Vertebral Augmentation System, DFine Europe), which allows the physician to pause cement application, reposition the needle if necessary, and resume without compromising the cement quality. However, careful monitoring of the cement distribution is essential, and patients must be informed about the potential neurological risks prior to the procedure.

The remote control also significantly reduces radiation exposure for the operator, even when using fluoroscopy or, preferably, single-slice CT imaging during cement application [29]. The advantages of RFS may facilitate the treatment of patients with pathological fractures. It is assumed that sealing the fracture lines or osteolytic areas leads to greater stability in the sacrum, thereby reducing pain. Based on our experience with different sacral cement augmentation techniques, patient hospitalization can be minimized if the fracture lines or destructed bone are adequately sealed. In our case, the patient was discharged 2 days after the procedure with significantly reduced pain, and the planned radiation therapy was not delayed. As a minimally invasive interventional pain therapy, RFS presents a promising treatment option for patients with metastatic bone infiltration or destruction, particularly in the context of multidisciplinary care for patients with severe pain and reduced mobility.

## Conclusion

RFS is an effective therapeutic option within the broader palliative care framework for patients suffering from tumor-related sacral destruction. The use of viscous cement minimizes the risk of leakage in areas of bone defects, making RFS a safe and straightforward procedure. It offers substantial pain relief and significantly enhances the patient’s quality of life. However, in cases where RFS technology is not accessible, the preferred approach should be the method that offers the highest level of clinical expertise.

## References

[1] Roodman GD . Mechanisms of bone metastasis. N Engl J Med 2004;350:1655–64. 10.1056/NEJMra030831. 15084698

[2] Siegel RL, Miller KD, Jemal A. Cancer statistics, 2018. CA Cancer J Clin 2018;68:7–30. 10.3322/caac.21442. 29313949

[3] Hess GM . Sacroplasty for the treatment of sacral insufficiency fractures. Unfallchirurg 2006;109:681–6. 10.1007/s00113-006-1102-9. 16897023

[4] Frey ME, DePalma MJ, Cifu DX, et al. Efficacy and safety of percutaneous sacroplasty for painful osteoporotic sacral insufficiency fractures: a prospective, multicenter trial. Spine 2007;32:1635–40. 10.1097/BRS.0b013e318074d4e1. 17621211

[5] Andresen R, Radmer S, Wollny M, et al. CT-guided cement sacroplasty (CSP) as pain therapy in non-dislocated insufficiency fractures. Eur J Orthop Surg Traumatol 2017;27:1045–50. 10.1007/s00590-017-2001-1. 28653101 PMC5686249

[6] Yoong J, Chandra RV, William L, et al. Percutaneous Sacroplasty for painful bone metastases: a case report. Pain Pract 2017;17:945–51. 10.1111/papr.12538. 27910200

[7] Tian QH, Liu HF, Wang T, et al. Percutaneous sacroplasty for painful sacral metastases involving multiple sacral vertebral bodies: initial experience with an interpedicular approach. Korean J Radiol 2019;20:939–46. 10.3348/kjr.2018.0803. 31132819 PMC6536795

[8] Tian QH, Liu HF, Wang T, et al. Fluoroscopy-guided percutaneous Sacroplasty for painful metastases at the sacral ala. J Pain Res 2020;13:151–6. 10.2147/JPR.S193866. 32021404 PMC6970629

[9] Tian QH, Han K, Wang T, et al. Percutaneous sacroplasty with or without radiofrequency ablation for treatment of painful sacral metastases. Am J Neuroradiol 2022;43:1222–7. 10.3174/ajnr.A7587. 35863777 PMC9575424

[10] Andresen R, Radmer S, Andresen JR, et al. Clinical improvement and cost-effectiveness of CT-guided radiofrequency Sacroplasty (RFS) and cement Sacroplasty (CSP) - a prospective randomised comparison of methods. Z Orthop Unfall 2019;157:524–33. 10.1055/a-0815-5073. 30736085

[11] Andresen R, Radmer S, Kamusella P, et al. Treatment of Denis 1, 2 and 3 insufficiency fracture zones of the os sacrum. Individual approaches adapted to the course of the fracture in CT-assisted balloon sacroplasty. *Osteologie* (Osteology) 2012;21:168–73. 10.1055/s-0037-1621680.

[12] Dehdashti AR, Martin JB, Jean B, et al. PMMA cementoplasty in symptomatic metastatic lesions of the S1 vertebral body. Cardiovasc Intervent Radiol 2000;23:235–7. 10.1007/s002700010052. 10821903

[13] Hierholzer J, Anselmetti G, Fuchs H, et al. Percutaneous osteoplasty as a treatment for painful malignant bone lesions of the pelvis and femur. J Vasc Interv Radiol 2003;14:773–7. 10.1097/01.rvi.0000079987.80153.85. 12817045

[14] Butler CL, Given CA 2nd, Michel SJ, et al. Percutaneous sacroplasty for the treatment of sacral insufficiency fractures. Am J Roentgenol 2005;184:1956–9. 10.2214/ajr.184.6.01841956. 15908561

[15] Uemura A, Matsusako M, Numaguchi Y, et al. Percutaneous sacroplasty for haemorrhagic metastases for hepatocellular carcinoma. Am J Neuroradiol 2005;26:493–5. 15760854 PMC7976465

[16] Masala S, Konda D, Massari F, et al. Sacroplasty and iliac osteoplasty under combined CT and fluoroscopic guidance. Spine 2006;31:E667–9. 10.1097/01.brs.0000231962.04739.ac. 16915083

[17] Atalay B, Caner H, Yilmaz C, et al. Sacral kyphoplasty for relieving pain caused by sacral hemangioma. Spinal Cord 2006;44:196–9. 10.1038/sj.sc.3101829. 16151449

[18] Valencia-Anguita J, Juliá-Narváez M, Rodríguez-Burgos F, et al. Ponce de León a. [Percutaneus sacroplasty for relieving pain caused by sacral metastases]. *Neurocirugica* (Astur) 2007;18:247–9 Article in Spanish. 10.4321/s1130-14732007000300009. 17622465

[19] Wee B, Shimal A, Stirling AJ, et al. CT-guided sacroplasty in advanced sacral destruction secondary to tumour infiltration. Clin Radiol 2008;63:906–12. 10.1016/j.crad.2008.02.010. 18625356

[20] Zhang J, Wu CG, Gu YF, et al. Percutaneous sacroplasty for sacral metastatic tumors under fluoroscopic guidance only. Korean J Radiol 2008;9:572–6. 10.3348/kjr.2008.9.6.572. 19039277 PMC2627246

[21] Georgy BA . Percutaneous cement augmentations of malignant lesions of the sacrum and pelvis. Am J Neuroradiol 2009;30:1357–9. 10.3174/ajnr.A1574. 19359653 PMC7051572

[22] Basile A, Tsetis D, Cavalli M, et al. Sacroplasty for local or massive localization of multiple myeloma. Cardiovasc Intervent Radiol 2010;33:1270–7. 10.1007/s00270-009-9761-x. 19967372

[23] Toro A, Pulvirenti E, Manfrè L, et al. Sacroplasty in a patient with bone metastases from hepatocellular carcinoma. A case report. Tumori 2010;96:172–4. 10.1177/030089161009600130. 20437879

[24] Lüdtke CW, Kamusella P, Andresen R. Pain management in pathologic sacrum fracture with CT guided balloon sacral vertebroplasty. Rofo 2012;184:578–80. 10.1055/s-0031-1299370. 22434369

[25] Shah RV . Sacral kyphoplasty for the treatment of painful sacral insufficiency fractures and metastases. Spine J 2012;12:113–20. 10.1016/j.spinee.2012.01.019. 22405614

[26] Sun G, Jin P, Li M, et al. Three-dimensional C-arm computed tomography reformation combined with fluoroscopic-guided sacroplasty for sacral metastases. Support Care Cancer 2012;20:2083–8. 10.1007/s00520-011-1317-3. 22081116

[27] Kortman K, Ortiz O, Miller T, et al. Multicenter study to assess the efficacy and safety of sacroplasty in patients with osteoporotic sacral insufficiency fractures or pathologic sacral lesions. J Neurointerv Surg 2013;5:461–6. 10.1136/neurintsurg-2012-010347. 22684691

[28] Pereira LP, Clarençon F, Cormier E, et al. Safety and effectiveness of percutaneous sacroplasty: a single-centre experience in 58 consecutive patients with tumours or osteoporotic insufficient fractures treated under fluoroscopic guidance. Eur Radiol 2013;23:2764–72. 10.1007/s00330-013-2881-3. 23689309

[29] Andresen R, Radmer S, Lüdtke CW, et al. Balloon sacroplasty as a palliative pain treatment in patients with metastasis-induced bone destruction and pathological fractures. Rofo 2014;186:881–6. 10.1055/s-0033-1356418. 24557599

[30] Dmytriw AA, Talla K, Smith R. Percutaneous sacroplasty for the management of painful pathologic fracture in a multiple myeloma patient: case report and review of the literature. Neuroradiol J 2017;30:80–3. 10.1177/1971400916678642. 27888274 PMC5564345

[31] Tarawneh AM, Sabou S, AlKalbani S, et al. Clinical outcomes of sacroplasty for metastatic sacral tumours: a systematic review and meta-analysis. Eur Spine J 2020;29:3116–22. 10.1007/s00586-020-06562-w. 32772170

[32] Prokop A, Andresen R, Chmielnicki M. Balloonsacroplasty: C-arm or CT controlled application?: experience with 46 patients. Unfallchirurg 2016, 119:929–35. 10.1007/s00113-015-2738-0. 25681131

[33] Andresen JR, Radmer S, Prokop A, et al. Diagnostics and treatment of sacral insufficiency fractures with special attention to cement augmentation procedures - an overview. Osteologie (Osteology) 2021;30:163–72. 10.1055/a-1154-9185.

